# Protective effects of Shensuitongzhi formula on intervertebral disc degeneration via downregulation of NF-κB signaling pathway and inflammatory response

**DOI:** 10.1186/s13018-023-04391-3

**Published:** 2024-01-19

**Authors:** Xu Wang, Qinghe Zeng, Qinwen Ge, Songfeng Hu, Hongting Jin, Ping-er Wang, Ju Li

**Affiliations:** 1 Institute of Orthopedics and Traumatology, The First Affiliated Hospital of Zhejiang Chinese Medical University (Zhejiang Provincial Hospital of Chinese Medicine), Hangzhou, 310006 China; 2 The First College of Clinical Medicine, Zhejiang Chinese Medical University, Hangzhou, 310053 China; 3 Department of Orthopaedics and Traumatology, Shaoxing Hospital of Traditional Chinese Medicine, Shaoxing, 312000 Zhejiang China; 4 Department of Orthopaedic Surgery, The First Affiliated Hospital of Zhejiang Chinese Medical University, Hangzhou, 310006 China

**Keywords:** Intervertebral disc degeneration, Shensuitongzhi formula, Lumbar spine instability surgery, Cartilage endplate calcification, NF-κB signaling pathway

## Abstract

Low back pain (LBP) is a common orthopedic disease over the world. Lumbar intervertebral disc degeneration (IDD) is regarded as an important cause of LBP. Shensuitongzhi formula (SSTZF) is a drug used in clinical treatment for orthopedic diseases. It has been found that SSTZF can have a good treatment for IDD. But the exact mechanism has not been clarified. The results showed that SSTZF protects against LSI-induced degeneration of cartilage endplates and intervertebral discs. Meanwhile, SSTZF treatment dramatically reduces the expression of inflammatory factor as well as the expression of catabolism protein and upregulates the expression of anabolism protein in LSI-induced mice. In addition, SSTZF delayed the progression of LSI-induced IDD via downregulation the level of NF-κB signaling key gene RELA and phosphorylation of key protein P65 in endplate chondrocytes. Our study has illustrated the treatment as well as the latent mechanism of SSTZF in IDD.

## Introduction

Low back pain (LBP) is a common orthopedic disease over the world and has become the greatest cause of disability globally [12], resulting in huge financial and social burdens [13]. About 540 million patients are given the diagnostic code reflective of spinal osteoarthritis. LBP is defined as pain and discomfort located to lumbar region and/or gluteal region, anatomically from the 12th thoracic vertebra to the gluteal sulcus with or without radiating pain [14]. In most cases, it is difficult to identify the specific structural cause of pain [15]. At present, surgery has not have a satisfactory long-term effect despite the main clinical treatment for IDD. Based on this, many studies turn to non-invasive alternatives for IDD [16]. More and more Chinese medicines have been dug that they have an amazing treatment on IDD [17–20]. Shensuitongzhi formula (SSTZF) is a drug commonly used in clinical treatment for orthopedic diseases such as fracture [21], osteoarthritis [21] and osteoporosis. It has been suggested that IDD shows similarity changing in cartilage with articular cartilage degeneration [20]. At the same time, it has been found that SSTZF can also have a good treatment for IDD [22]. But the exact mechanism has not been clarified, which limits the further development and application of SSTZF.

LBP is closely related to lifestyle and living conditions, such as obesity, poor sleep, stress and smoking [23]. The etiology of LBP remains unclearly. Extensive imaging and histopathology studies have found that lumbar intervertebral disc degeneration (IDD) is an important cause of LBP [24]. Physiologically, the intervertebral disc (IVD), an important part of the spine [25], consists of three major tissues: nucleus pulposus (NP), cartilaginous endplate (CEP) and annulus fibrosus (AF) [26]. The pathogenesis of IDD has not been fully elucidated which is characterized as losing and producing insufficiency of extracellular matrix (ECM) because of the decrease in number and function of NP cells [27, 28]. Type II collagen (Col II) and proteoglycan (PG) are the main components in ECM [16]. In the process of IDD, there are not only an obvious imbalance between the anabolism and catabolism of ECM [11] but also producing a large number of inflammatory factors such as TNF-α and IL-1β which promote the reduction of matrix by directly inhibiting the expression of matrix genes or by increasing the expression of collagen and aggregator lyase [29].

Previous studies have mainly focused on the nucleus pulposus which is the core structure of IVD and shows obvious changes in X-ray, while changes in the cartilage endplate are rarely reported [30, 31]. CEP is a thin layer of hyaline cartilage that not only separates the vertebral body from disc [32, 33], but also residues mechanical forces from intravertebral disc pressure [34]. The CEP is a semi-permeable barrier by which nutrients entering the disc and metabolites expelled. Therefore, CEP degeneration not only changes the mechanical function of CEP, but also affects the bio-transport function of CEP [35, 36]. Chondrocyte apoptosis and endplate calcification are considered as two decisive manifestations of cartilage endplate degeneration [37, 38]. Therefore, protection against CEP degeneration is a potential treatment strategy for maintaining IVD health and preventing spinal disease.

The nuclear factor-κB (NF-κB) signaling pathway plays a key regulatory role in inflammatory response and immune response [31]. The importance of NF-κB signaling pathway in the cause and treatment of IDD has been reported in many studies. For example, Wang et al. [39] found that the activation of NF-κB can promote the overexpression of MMPs and then lead to the over-decomposition of ECM, thus leading to IDD. Meanwhile, NF-κB signaling pathway activation can increase many inflammatory mediators and chemokine expression levels, resulting in a vicious cycle that further accelerates the progression of IDD [9, 30, 40]. Studies using losing function approaches confirmed the importance of NF­κB in IDD [40, 41]. In addition, a large number of studies have found that TCM which has efficacy on tonifying kidney and promoting blood circulation can alleviate IDD through NF-κB pathway [42]. Many studies have focused on the action mechanism of NF-kB in NP [30, 31]. However, the effect of NF-κB signaling on cartilage endplates is unknown.

Based on the above-mentioned studies, we hypothesized that SSTZF could ameliorate the pathological changes in inflammation-associated chondrocytes with potential drug therapy for IDD. In the present study, we evaluated the therapeutic effect of SSTZF on IDD and investigated the potential mechanism of action with in vivo and in vitro experiments.

## Materials and methods

### Preparation of SSTZF

SSTZF whole formula consists of rehmanniae, eucommia, saponaria, wolfberry, cinnamon, cornelian, peach kernel, safflower, yam and licorice (Table 1). Specific concoction method is divided into two parts: water extraction and alcohol extraction. Water extraction part: firstly eight Chinese herbal beverages of rehmanniae, eucommia, saponaria, wolfberry, cornelian, safflower, yam and licorice were soaked in pure water for 1 h, and 12 times the volume of pure water was added to the Chinese herbal beverages for reflux extraction, for a total of three times, 1.5 h each time. Then, 95% ethanol (final alcohol content of 60%) was added to alcohol precipitation for 24 h, and ethanol was recovered under reduced pressure. Alcohol extraction part: soak cinnamon and peach kernel in 10 times the volume of 60% ethanol for 1 h, extract at reflux for three times, 1.5 h each time, and recover the ethanol under reduced pressure. Finally, the extracts of the above two parts were combined and concentrated under reduced pressure to a raw drug concentration of 2 g/mL. The prepared herbs were stored in -30℃ refrigerator for subsequent experiments.

**Table 1:** Compositions of SSTZF

Chinese name	Botanical name	Common name	Parts used	Proportion (%)
Tao ren	Prunus persica (Batsch.)	Peach	Fruit	11.8
Rou gui	Cinnamomum cassia (Presl)	Cassia Bark Tree	Bark	5.9
Fu zi	Aconitum carmichaeli (Debx.)	Monkshood	Root	11.8
Du zhong	Eucommia ulmoides (Oliv.)	Eucommia	Bark	11.8
Gou qi	Lycium barbarum (L.)	Matrimony Vine	Fruit	11.8
Shan yao	Dioscoreae opposite (Thunb.)	Common Yam	Root	11.8
Hong hua	Carthamus tinctorius (L.)	False Saffron	Corolla	5.9
Shan zhu yu	Cornus officinalis (Sieb.)	Medical Dogwood	Fruit	5.9
Gan cao	Glycyrrhiza uralensis (Fisch.)	Ural Licorice	Root	5.9
Shu di huang	Rehmannia glutinosa(Liboscb)	Rehmannia	Root	17.4

### Preparation of SSTZF-containing serum

Twenty SD rats were randomly divided into two groups, blank group and SSTZF group, and the dose was converted with reference to the ratio of body surface area of experimental rats to that of adults, once daily for 7 days, and the blank group was given an equal amount of distilled water. The blood was collected from the abdominal aorta 1 h after the last dose, left to stand for 1 h and then centrifuged at 3000 rpm/min for 10 min. Finally, the upper serum was aspirated for complement extinguishing, filtered, sterilized and then stored at − 80 ℃.

### Experimental animals

Ten-week-old male C57BL/6 J mice were provided by the Animal Center of Zhejiang University of Traditional Chinese Medicine (Hangzhou, China). All mice were housed in the pathogen-free laboratory of the Animal Center of Zhejiang University of Traditional Chinese Medicine (Hangzhou, China), with six mice per cage and free access to clean food and water. All experiments were conducted after approval by the Animal Experimentation Ethics Committee of Zhejiang University of Traditional Chinese Medicine (LZ12H27001).

### Experimental grouping and drug administration treatment

The experimental mice were randomly divided into sham group, model group and SSTZF group, in which mice in both model and SSTZF groups underwent lumbar disc degeneration model surgery. Twelve mice in each group were sampled at 4 weeks and 8 weeks postoperatively, six mice in each time point. According to previous studies, the lumbar degeneration surgery was taken to induce IDD in the lumbar instability mouse model [1, 2]. Briefly, pentobarbital sodium-anesthetized mice were positioned prone, and the supraspinous ligament, interspinous ligament and spinous process between their second lumbar and sixth lumbar vertebrae were freed, and then, the supraspinous ligament, interspinous ligament and spinous process between the third and fifth lumbar vertebrae were clipped with curved scissors. Meanwhile, only the paravertebral muscle tissue was removed from the mice in the sham group. Finally, the incision was sutured and antibiotics were administered to prevent infection and put back into the mouse cage.

### Micro-CT(μCT) analysis

Spinal tissues were collected from the thirteenth thoracic vertebra to the first sacral vertebra of mice after 4 weeks and 8 weeks postoperatively, and the muscle tissues around the spine were stripped clean and then fixed in 4% cellular tissue fixative for three days. The fixed end tissue samples were placed in the micro-CT mouse bed in the sagittal direction for X-ray scanning. After scanning, 3D reconstruction and 3D image creation were performed using on-board NRecon reconstruction software and CTvox graphics software to observe the lumbar disc tissue gap size and endplate ossification formation. Finally, the region of interest (ROI) was quantitatively analyzed using the airborne bone micromorphometric analysis software CTAn [1].

### Histomorphological staining

After rinsing the residual fixative from the surface with running water at the end of fixation, the samples were submerged in 14% EDTA solution for 14 days for decalcification. Then, the surface decalcification solution was rinsed under running water and placed in gradient alcohol for dehydration and subsequent paraffin embedding. Paraffin tissues were stained in sections of 3 μm thickness. After the sections were dewaxed and rehydrated, the lumbar disc tissues were morphologically observed with Alcian blue/hematoxylin and orange G staining (ABH staining). Based on the results of ABH staining and the histological assessment scale for lumbar disc tissue, data were evaluated for each histological variable in the disc structure and cartilage endplate structure, respectively [3].

### Tartrate-resistant acid phosphatase (TRAP) staining

Tissue sections from each experimental group were stained by tartrate-resistant acid phosphatase (TRAP) to detect the activity of osteoclasts in the endplate of the lumbar disc cartilage in mice and to quantify the number of osteoclasts (N.Oc/TA) as previously described. Briefly, anhydrous sodium acetate, sodium tartrate and glacial acetic acid were prepared into a 200-mL base working solution and preheated at 37 °C. The slices were dewaxed and rehydrated into the base working solution with naphthol AS-BI phosphate and baked at 37 °C for 1 h. Then, transfer to another base working solution mixed with sodium nitrite and basic magenta and incubate for 5–10 min; the color appears to terminate the staining. Finally, alcohol-free hematoxylin staining was used as the background color.

### Immunohistochemical staining

For immunohistochemical (IHC) assays, the sections were treated with 0.01 M citrate buffer (Solarbio, Beijing, China) for 4 h at 60 °C as antigen repair after dewaxing and rehydration was completed. Next, the sections were incubated in Col2, Mmp13, IL-1β, TNF-α and p-P65 primary antibodies overnight at 4 °C. The next day after incubation with the cognate secondary antibody for 20 min, diaminobenzidine (DAB) solution was applied to detect positive staining, while hematoxylin was applied for counterstaining. Positive staining was assessed semi-quantitatively using Image-Pro Plus software (Media Cybernetics, Silver Spring, USA).

### Cell culture

Primary chondrocytes were obtained from two-week-old C57BL/6 J mice. The mice were executed and sterilized by immersion in 75% alcohol, and lumbar endplate cartilage was isolated aseptically. Then, phosphate buffer was washed three times and digested with 0.25% collagenase dissolved in F12/ DMEM medium in a cell culture incubator overnight. The following day, the digested primary chondrocytes were cultured in DMEM/F12 medium containing 10% FBS and 1% streptomycin/penicillin and incubated in an incubator at 37 °C and 5% CO2 for subsequent experiments.

#### Real-time quantitative PCR assay

Lumbar vertebral bone tissue stored in − 80 °C refrigerator was ground into powder form with liquid nitrogen, and then, a portion was added to 1 mL of Trizol solution. The primary chondrocytes cultured in vitro were added to 500μLTrizol solution. Bone tissue RNA was extracted and reverse transcribed according to the operating instructions of RNeasy Mini Kit (QIAGEN) and All-in-One cDNA Synthesis SuperMix (Bimake). The qPCR assay was then performed with 2 × SYBR Green qPCR Master Mix (Low ROX) (Bimake) reagent. And β-actin was used as a control gene for quantitative analysis. The target gene primer sequences were as follows:

**Table:** 

Primer name	Primer sequences (5’ → 3’)
*IL-1β* Forward	GCAACTGTTCCTGAACTCAACT
*IL-1β* Reverse	ATCTTTTGGGGTCCGTCAACT
*TNFα* Forward	CCCTCACACTCAGATCATCTTCT
*TNFα* Reverse	GCTACGACGTGGGCTACAG
*Mmp13* Forward	TTTGAGAACACGGGGAAGA
*Mmp13* Reverse	ACTTTGTTGCCAATTCCAGG
*Col2* Forward	TGGTCCTCT GGGCATCTCAGGC
*Col2* Reverse	GGTGAACCTGCTGTTGCCCTCA
*RELA* Forward	AGGCTTCTGGGCCTTATGTG
*RELA* Reverse	TGCTTCTCTCGCCAGGAATAC
*β-actin* Forward	GGAGATTACTGCCCTGGCTCCTA
*β-actin* Reverse	GACTCATCGTACTCCTGCTTGCTG

#### Western Blot assay

Another portion of the above ground powdered bone tissue and in vitro cultured primary chondrocytes were added to the appropriate amount of RIPA lysis solution (containing protease inhibitor, ready to use) and lysed by repeated shaking on ice for 30 min. Then, place in a centrifuge at 4 °C for 10 min at 12,000 rpm and aspirate the supernatant into a new 1.5-mL centrifuge tube. The protein concentration of each group was first measured by the BCA protein quantification kit, and then, a certain volume of 5 × loading buffer was added to the remaining protein supernatant (so that the final concentration was 1 ×), and finally denatured by boiling at 100 ℃ for 5 min. Next, proteins were separated on 8% SDS–PAGE gels (20 μg/lane) and transferred to NC membranes. After incubation with 5% skim milk for 1 h, the primary antibodies were incubated with Col2 (1:1000 dilution, Abcam), Mmp13 (1:1000 dilution, Huaan), p65 (1:1000 dilution, Cell Signaling Technology), p-P65 (1:1000 dilution, Cell Signaling Technology) and β-actin (1:10,000 dilution, Sigma-Aldrich) for 16 h at 4 °C. After that, the protein bands were visualized by incubation with the corresponding cognate secondary antibodies for 1 h at room temperature and finally with Image Quant LAS 4000 (EG, USA). Using β-actin as an internal reference, the grayscale values of the protein bands were calculated using ImageJ software to analyze the expression of the target protein between groups.

#### Statistical analysis

All experimental data in this subject were statistically analyzed using SPSS 25.0 software, and the statistical results of the measures were expressed as mean ± standard deviation. One-way analysis of variance (one-way ANOVA) was used for comparison between different groups, and if the variance was not equal, Dunnett's T3 test was used. The difference was statistically significant at *P* < 0.05.

## Results

### SSTZF attenuates cartilage endplate calcification and delays disc degeneration

**Fig. 1:**
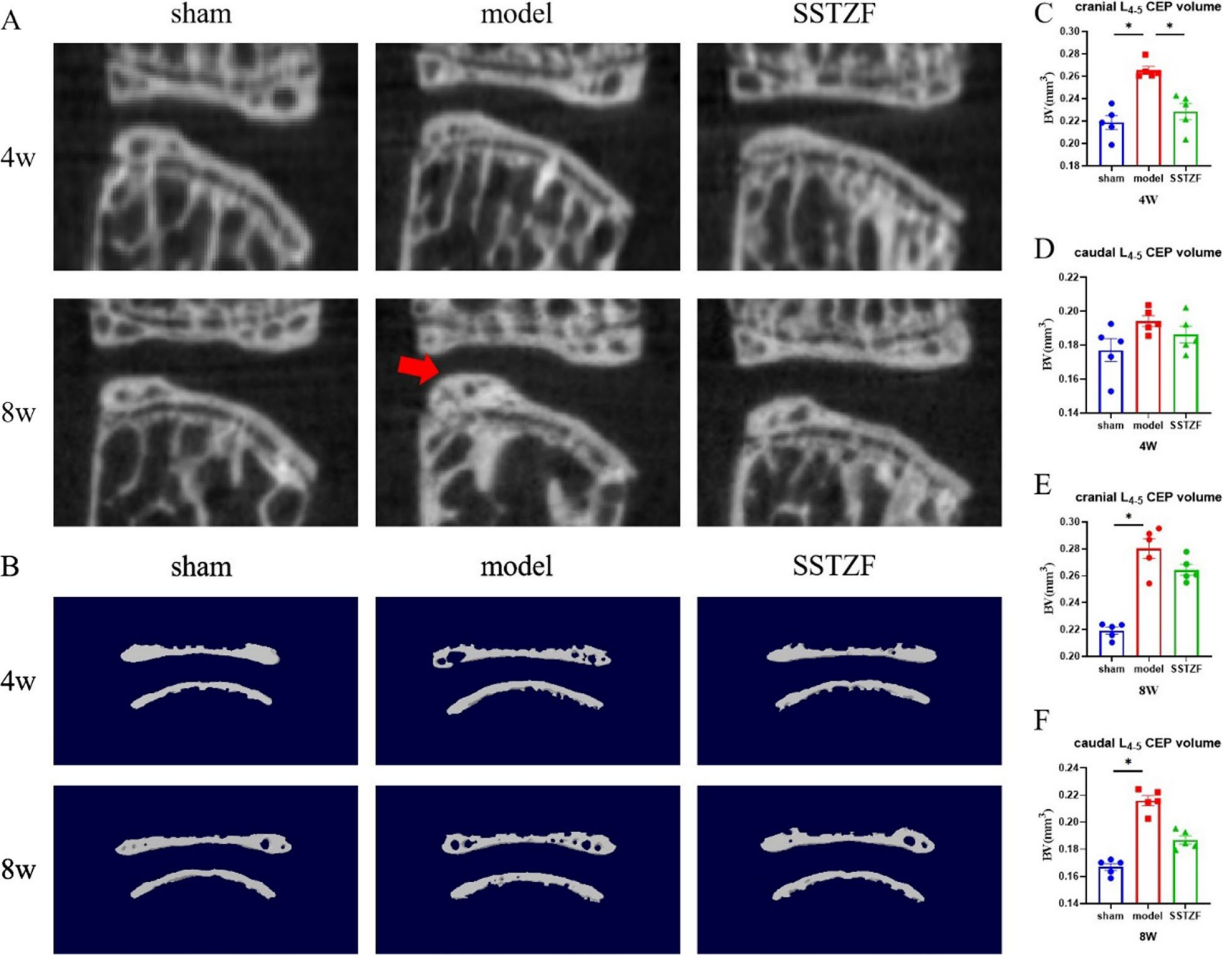
SSTZF attenuates disc degeneration in LSI-operated mice. **A** 2D image of the median sagittal view of L4-5 lumbar spine; the red shear head indicates the L4-5 intervertebral space; **B** 3D image of the cartilage endplate ossification layer of L4-5 lumbar spine; the red arrows indicate the ossification layer cavity structure; **C**, **D** the bone volume of the lower cartilage endplate ossification layer of L4 lumbar spine and the upper cartilage endplate ossification layer of L5 lumbar spine after 4 weeks of modeling; **E**, **F** the bone volume of the lower cartilage endplate ossification layer of L4 lumbar spine and the upper cartilage endplate ossification layer of L5 lumbar spine after 8 weeks of modeling, respectively. Data were presented as means ± S.D.*P < 0.05; **P < 0.01; ***P < 0.001; ns: no significant difference. n = 6 for each group

Narrowing the IVD space is one of the typical features of disc degeneration, so we first focus on the spatial variation in the lumbar interstitial space. Midsagittal scanning images indicated that the LSI operation narrowed disc height which between L4 and L5. However, the descending height could be partly rescued by SSTZF (Fig. 1A). In addition, the results of the three-dimensional (3D) reconstruction of disc and cartilage endplates in SSTZF are as same as the trend of intervertebral space size change (Fig. 1B). Also, the 3D reconstructions show that the cavity in the region of endplate ossification is the main cause of the reduction in disc volume, whereas the cartilage endplates are relatively intact in the sham-operated group mice.

Importantly, the bone volume in the region of endplate ossification was significantly reduced in mice at week 4 of SSTZF treatment compared with the model group especially in the L4 IVD (Fig. 1C and D). However, the bone volume in the endplate ossification region was reduced at week 8 of SSTZF treatment; there was no statistical difference compared with the model group (Fig. 1E and F). These data suggest that SSTZF can delay the degeneration of IDD in LSI mice.

### SSTZF protects against LSI-induced degeneration of cartilage endplates and intervertebral discs in mice

As the radiological results already suggest that SSTZF could inhibit cartilage endplate ossification in LSI mice, ABH staining was used to assess the degeneration of the intervertebral discs in LSI mice.

**Fig. 2:**
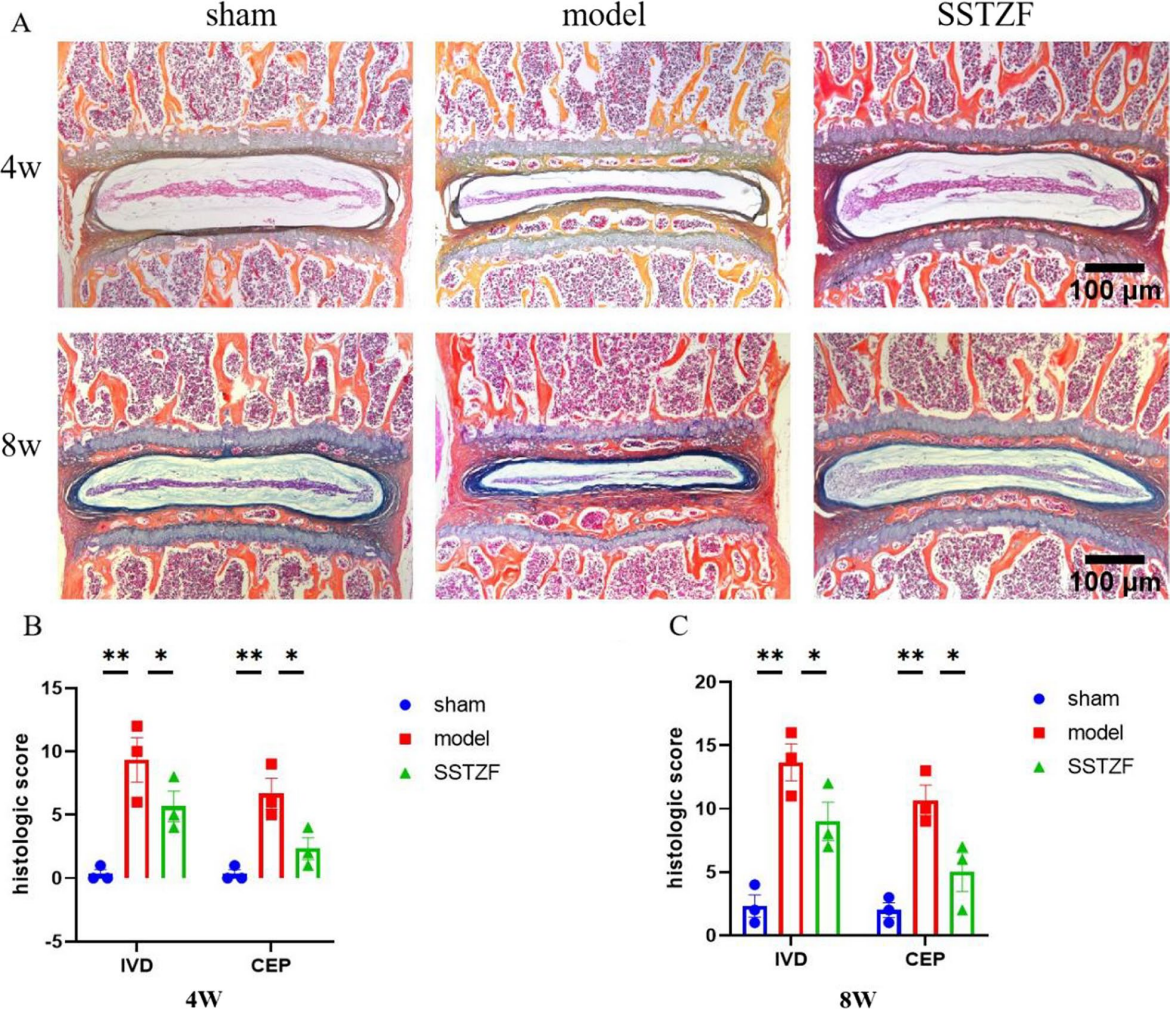
SSTZF attenuates cartilage endplate calcification in LSI mice. **A** ABH staining of the entire L4/5 intervertebral disc. **B** Histological variables scored in the disc region and cartilage endplate region in the lumbar spine of 4W mice after LSI surgery. **C** Histological variables scored in the disc region and cartilage endplate region in the lumbar spine of 8W mice after LSI surgery. IVD: disc region, CEP: cartilage endplate region. Data were presented as means ± S.D.*P < 0.05; **P < 0.01; ***P < 0.001; ns: no significant difference. n = 6 for each group

Compared with the sham group, the NP region of the intervertebral disc in the LSI group is significantly extruded and deformed severe volume reduction. The CEP region has formed obvious ossification centers with thickened ossification layers and obvious cavity structures which were gradually worsened with age (Fig. 2A).

**Fig. 3:**
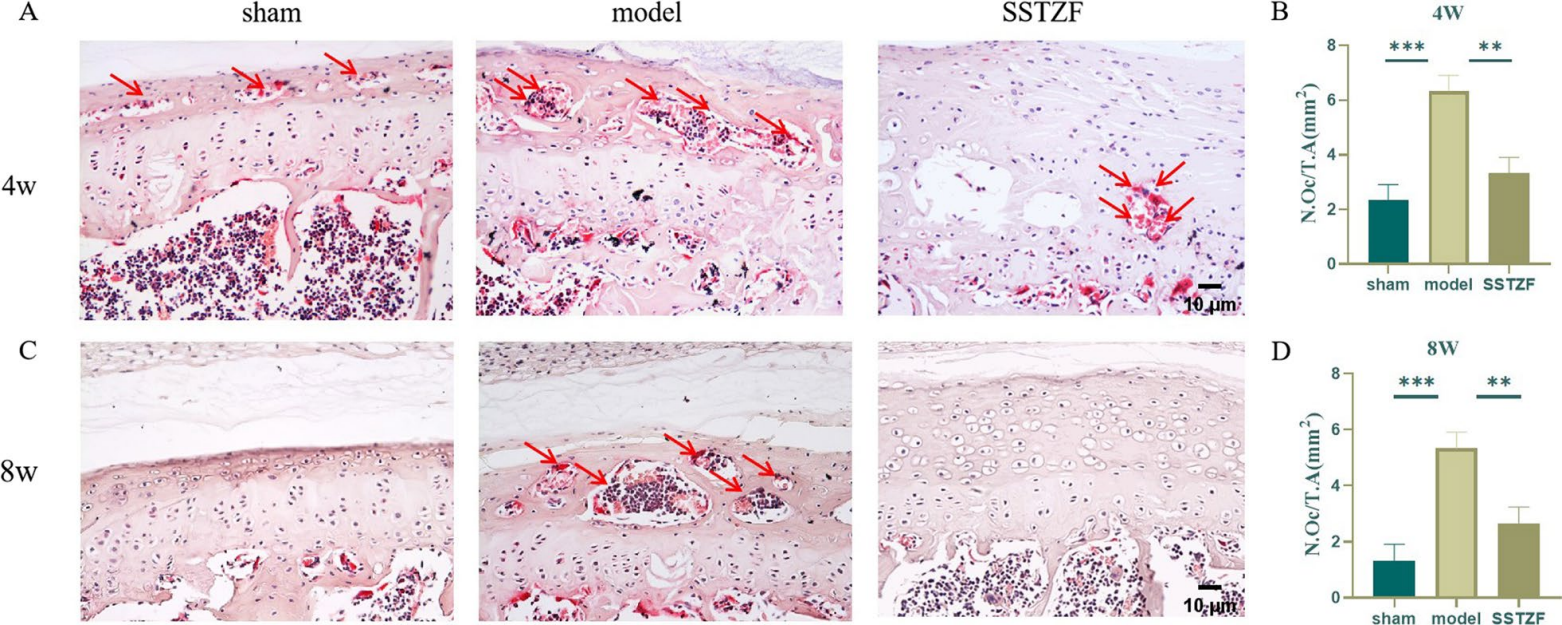
SSTZF attenuates osteoclast activity in the intervertebral discs of LSI mice. **A**, **B** Proportion of TRAP-stained and positive cells in the endplate of the disc cartilage of the lumbar spine of LSI-operated 4W mice. Red arrows indicate positive cells. **C**, **D** Proportion of TRAP staining and positive cells in the endplate of intervertebral disc cartilage in the lumbar spine of LSI-operated 8W mice. Red arrows indicate positive cells. Data were presented as means ± S.D.*P < 0.05; **P < 0.01; ***P < 0.001; ns: no significant difference. n > 3 for each group

Interestingly, SSTZF mice showed that the structure of NP was still relatively intact at 4 weeks and it arises a situation that little chondrocytes in CEP region showed hypertrophic differentiation. As well, the cartilage endplates were significantly delayed calcification at 8 weeks (Fig. 2A). These data suggest that SSTZF can improve the progression of LSI-induced IDD. In addition, disc and cartilage endplate scores are significantly lower in the SSTZF group than in the model group at both 4 and 8 weeks (Fig. 2B and C). Because the appearance of cavities in ossified areas of the cartilage endplates may cause by the increasing osteoclasts activity, we observed the degree of osteoclasts activity in mice by Trap staining. The results showed that osteoclasts are mostly active in the cartilage endplate region in model group during the process of IDD, especially in the cavity after cartilage endplate ossification. However, SSTZF treatment significantly inhibits osteoclasts secreting TRAP in cartilage endplate in mice (Fig. 3A–D). These above histopathological data suggest that SSTZF effectively protects against LSI-induced degeneration of cartilage endplates and intervertebral discs.

### SSTZF delays LSI surgery-induced degradation of cartilage matrix

**Fig. 4:**
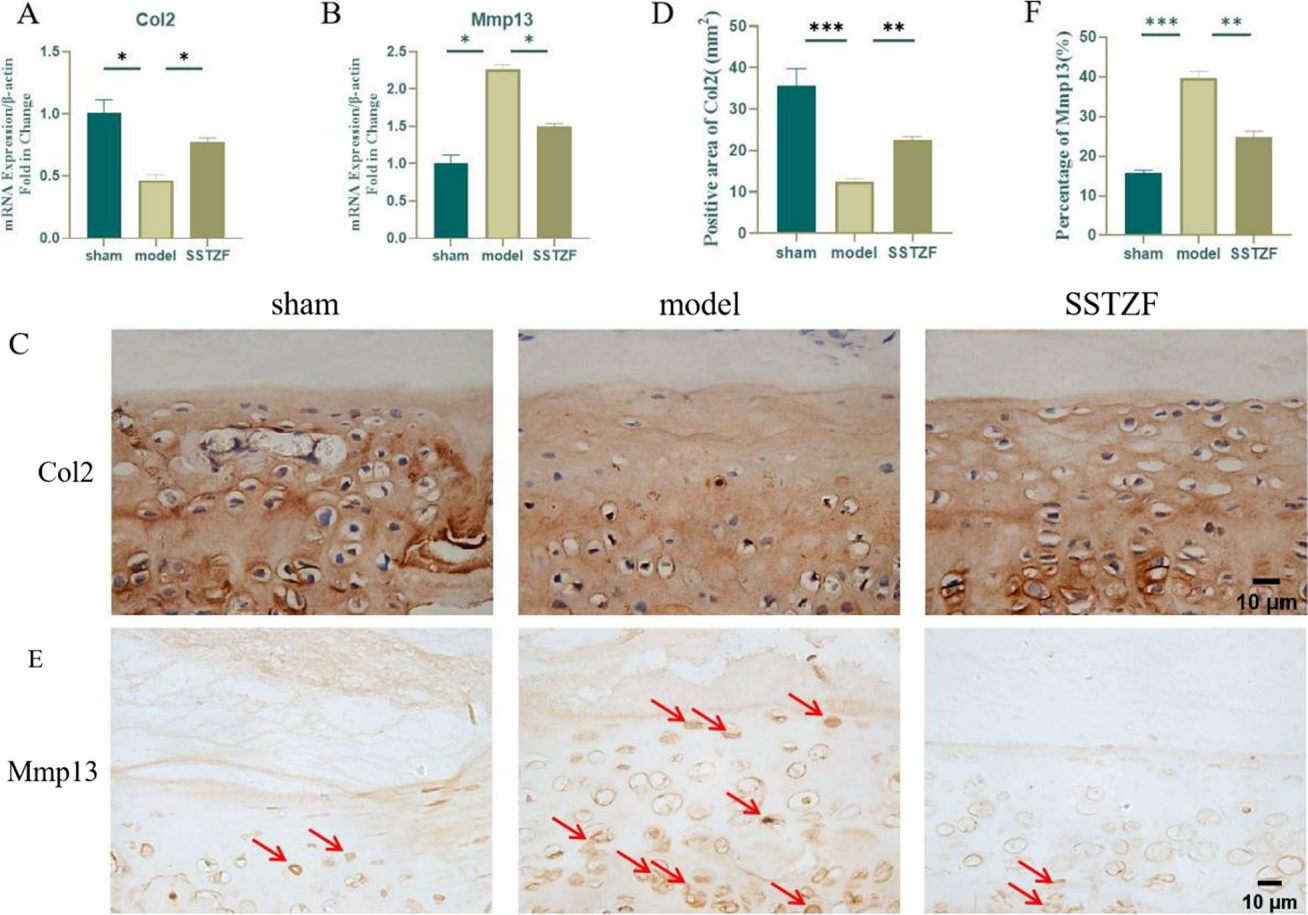
Effect of SSTZF on the anabolic and catabolic metabolism of cartilage endplates after LSI surgery. **A**, **B** Expression levels of Col2 and Mmp13 genes in bone tissue of mouse lumbar spine; **C**, **D** immunohistochemical staining and quantification of Col2 in the cartilage endplates of the intervertebral discs of mice with 8W LSI surgery; **E**, **F** immunohistochemical staining and percentage of positive cells of Mmp13 in the cartilage endplates of the intervertebral discs of mice with 8W LSI surgery. Red arrows indicate positive cells. Data were presented as means ± S.D.*P < 0.05; **P < 0.01; ***P < 0.001; ns: no significant difference. n > 3 for each group

The ECM is an important tissue structure of intervertebral disc which can withstand pressure. The CEP, as an important structural component of the disc, not only relieves the axial pressure on the NP, but also serves as a pathway for nutrient delivery to the internal tissues of the disc. First, the mRNA levels of anabolic/catabolic genes (Col2 and Mmp13) were detected in lumbar vertebral tissues of each group of mice by qRT-PCR. Compared with LSI group, Col2 expression was significantly increased in SSTZF group, while Mmp13 expression was greatly reduced (Fig. 4A and B). Then, the spatial expression was further examined by IHC which showed that Col2 expression was significantly reduced and Mmp13 expression was significantly increased in the intervertebral disc tissue in the model group (Fig. 4C–F). In contrast, SSTZF can significantly inhibit the downregulation of Col2 expression and elevation of Mmp13 expression in the intervertebral disc tissues of mice (Fig. 4C–F). These data suggest that SSTZF inhibits the degradation of cartilage matrix to delay IDD.

### SSTZF inhibits LSI-induced inflammatory factor expression

**Fig. 5:**
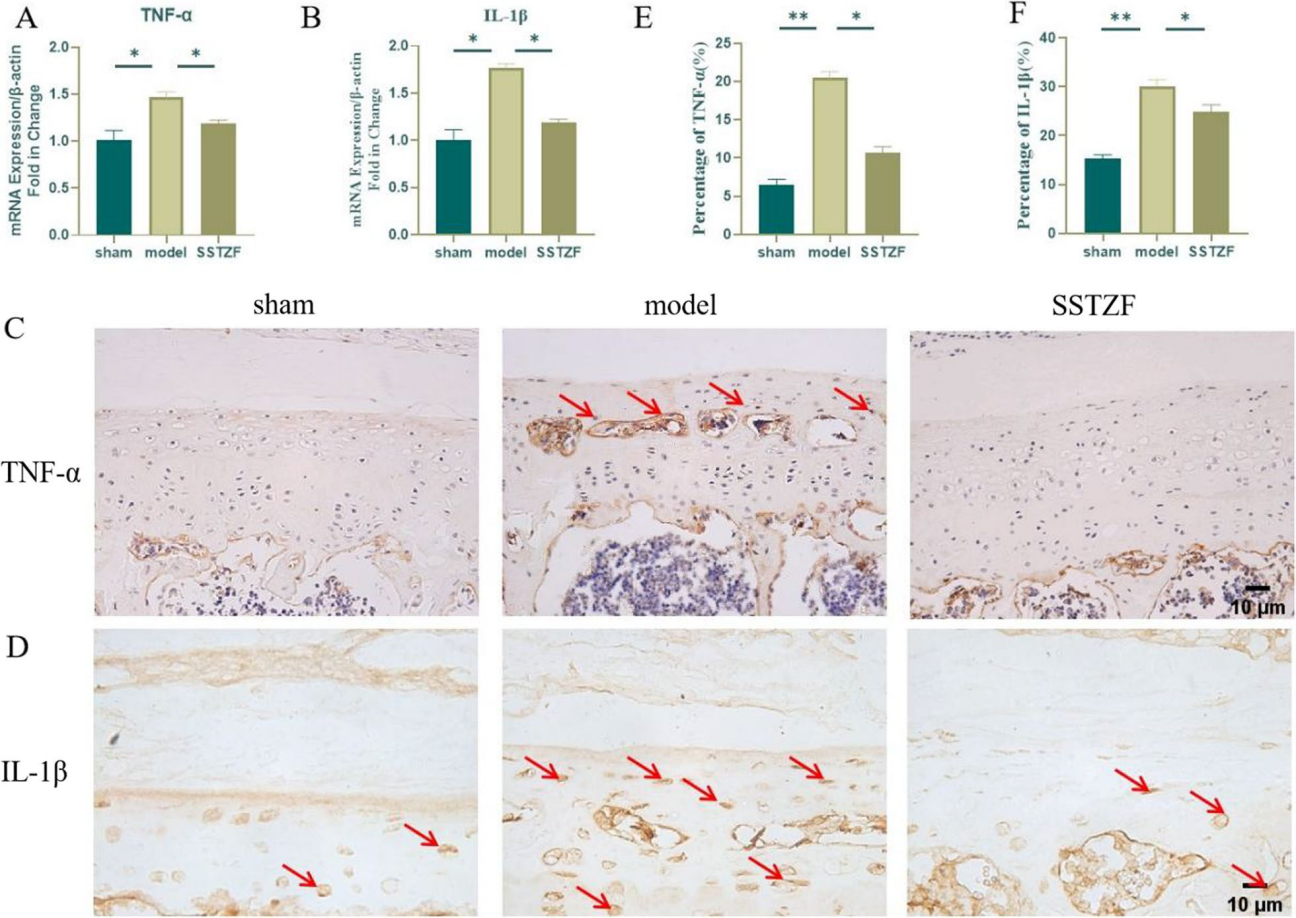
Effect of SSTZF on inflammatory factors in cartilage endplates after LSI surgery. **A**, **B** Expression levels of TNF-α and IL-1β genes in bone tissue of lumbar spine of mice; **C**, **D** immunohistochemistry of TNF-α and IL-1β in cartilage endplates of intervertebral discs of lumbar spine of mice with 8W LSI surgery. Red arrows indicate positive cells; **E**, **F** quantitative analysis of TNF-α and IL-1β positive cell rates. Data were presented as means ± S.D.*P < 0.05; **P < 0.01; ***P < 0.001; ns: no significant difference. n > 3 for each group

The involvement of inflammatory cytokines is one of the main factors leading to the degenerative disc changes as well as the imbalance of disc metabolism. The qRT-PCR analysis of lumbar spine bone tissue revealed that the mRNA expression of inflammatory factor genes such as IL-1β and TNF-α was significantly elevated in the lumbar spine of mice in the model group. However, SSTZF group was able to significantly reduce the expression of IL-1β and TNF-α (Fig. 5A and B). Then, we further examined their expression by IHC. Compared with the sham group, the results in the model group mice showed that a large number of TNF-α and IL-1β positive staining areas appeared in the cartilage endplate region of the lumbar discs, especially in the cartilage endplate cavity structure (Fig. 5C–F). However, SSTZF significantly inhibited the secretion of inflammatory factors (Fig. 5C–F). The above data suggest that SSTZF delays calcification and degeneration of the cartilage endplates by inhibiting the expression of inflammatory factors in LSI surgery-induced.

### SSTZF inhibits the activation of NF-κB signaling pathway in CEP to delay IDD

**Fig. 6:**
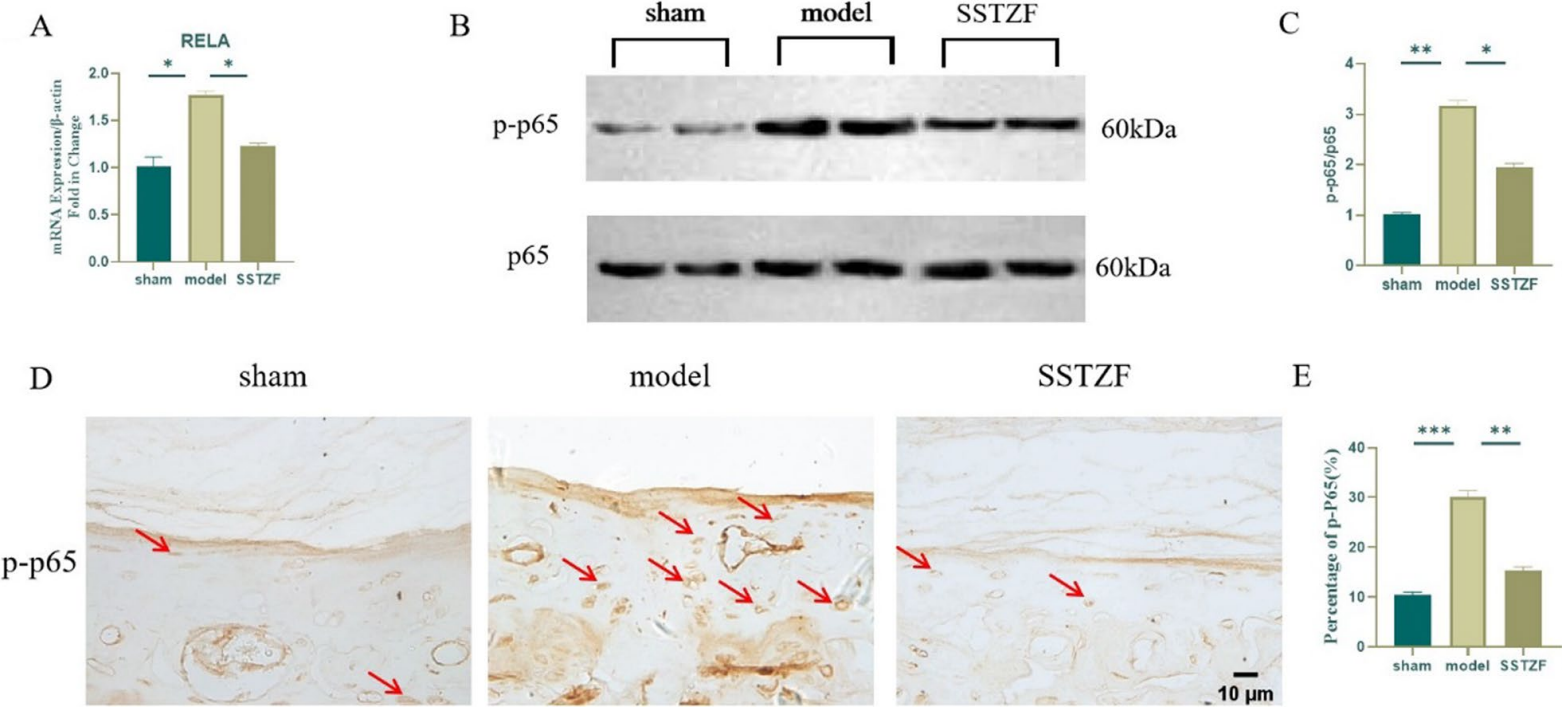
Effect of SSTZF on NF-κB signaling in cartilage endplates after LSI surgery. **A** Expression level of RELA gene in bone tissue of mouse lumbar spine; **B**, **C** protein expression level and quantitative analysis of p-p65 in bone tissue of mouse lumbar spine; **D**, **E** immunohistochemistry and quantitative analysis of positive cell rate of p-p65 in cartilage endplates of intervertebral discs of lumbar spine in mice with 8W LSI surgery. Data were presented as means ± S.D.*P < 0.05; **P < 0.01; ***P < 0.001; ns: no significant difference. n > 3 for each group

Studies have shown that IL-1β and TNF-α (two inflammatory cytokines) closely associate with IDD as well as closely link to the NF-κB signaling pathway [4–11]. However, whether SSTZF inhibits NF-κB signaling in cartilage endplates has not been clarified. First, qRT-PCR analysis of lumbar vertebral bone tissue revealed that the expression level of RELA, a key regulatory gene of NF-κB signaling, was significantly inhibited by SSTZF compared with the model group (Fig. 6A). Then, we detected the expression of p-p65, a key regulatory protein of NF-κB signaling pathway, in lumbar vertebral bone tissue by WB. As expected, SSTZF group significantly inhibited p-p65 protein expression in lumbar vertebral bone tissue of LSI group (Fig. 6B and C). Similarly, the SSTZF significantly inhibited p-p65 protein positive staining cells in the cartilage endplate region compared to the model group (Fig. 6D and E). These data suggest that SSTZF delaying IDD process is closely associated with the inhibition of NF-κB signaling pathway.

### SSTZF abrogates TNF-α treatment-induced activation of NF-κB signaling pathway, catabolism and inflammation in chondrocytes in vitro

**Fig. 7:**
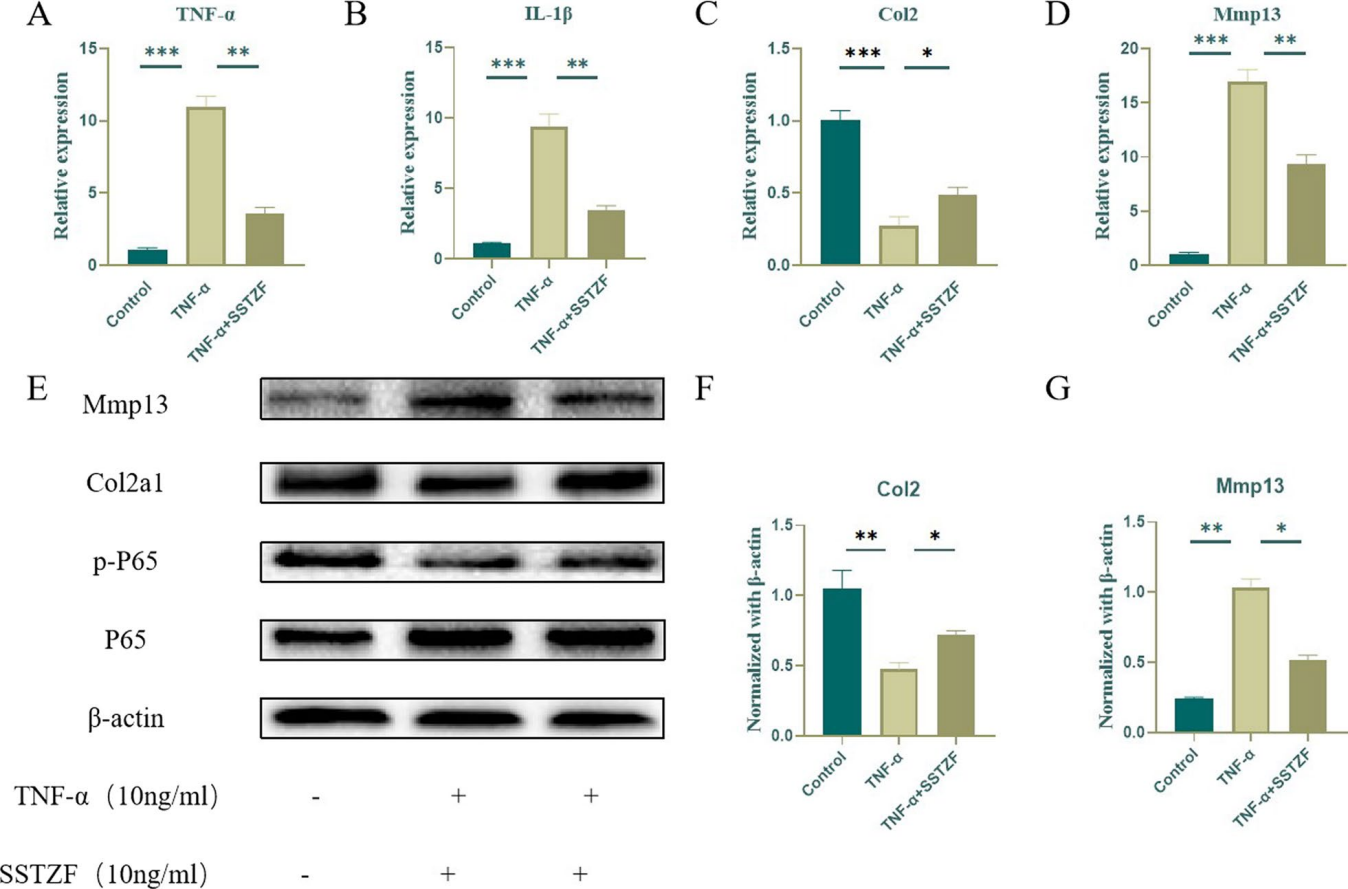
Effect of SSTZF on TNF-α-induced primary chondrocytes in vitro. **A**–**D** Gene expression of TNF-α-induced primary chondrocytes treated with SSTZF-containing serum for 24 h. **E**–**G** Protein expression of TNF-α-induced primary chondrocytes treated with SSTZF-containing serum for 24 h. Data were presented as means ± S.D.*P < 0.05; **P < 0.01; ***P < 0.001; ns: no significant difference. n = 3 for each group

The above data suggest that SSTZF can alleviate the degeneration of chondrocytes in endplate in vivo. Therefore, we used primary chondrocytes to investigate the effect of SSTZF on TNF-α-treated cells in vitro. First, a qRT-PCR assay showed that SSTZF significantly decreased the mRNA expression of Mmp13, IL-1β and TNF-α but increased the expression of Col2, which was consistent with the trend of IHC results in vivo (Fig. 7A–D). Then, WB was used to determine the changes of the expression levels of Col2, Mmp13 and p-p65 which is the key factor of NF-κB signaling pathway. The data showed that the protein expression levels of Mmp13 and p-p65 were significantly increased in TNF-α-treated chondrocytes, while the expression level of Col2 was significantly decreased. However, SSTZF-containing serum was able to inhibit the expression of Mmp13 and p-p65 and promote the expression of Col2 (Fig. 7E–G). These data partially confirm that SSTZF can protect chondrocytes from catabolic and inflammatory effects by inhibiting the activation of NF-κB signaling pathway.

## Discussion

For the first time, we proof the effect of SSTZF on the progression of IDD in animal models and explore its mechanism preliminarily. Histological and μCT results showed that SSTZF effectively delayed the degradation of cartilage matrix, inhibited the expression of LSI-induced inflammatory factors and protected the cartilaginous endplate calcification and intervertebral disc degeneration of LSI-induced mice. SSTZF was also demonstrated to delay IDD progression by inhibiting NF-κB signaling pathway in vivo and in vitro. These data provides a new and reliable evidence for the clinical treatment of LBP by SSTZF.

SSTZF, a traditional Chinese Herbal formula, has many function on different disease not only orthopedic disease but also internal disease [43].Our previous study had demonstrated that SSTZF may decelerate osteoarthritic cartilage degeneration [44] via reducing the expressional level of MMP13 in cartilage of DMM-induced mice [45]. In addition, it was been reported that had an effective treatment on fracture [21]. The recent results showed that SSTZF modulates NP cell proliferation and ECM remodeling in IDD [30]. As the same results as we has, in our experiment, SSTZF has the also function on IDD through restoring the metabolic balance of ECM. In this study, we used the LSI mouse model to simulate the human IDD process. μCT and ABH staining results were similar to those previously reported [46]. Reduced disc height, vertebral endplate sclerosis and decreased IVD volume were observed in LSI mice. However, mice treated with SSTZF showed the opposite result to the model group at 4 and 8 weeks after LSI surgery, suggesting that SSTZF delays disc degeneration and IDD progression by reducing cartilaginous endplate calcification.

Recent studies have shown that cartilage endplate degeneration is also a key factor in the pathogenesis of IDD [47]. The CEP is a thin layer of hyaluronic cartilage that acts as a semi-permeable barrier which not only delivers nutrients but also be a cushion for mechanical load [33]. In the IDD process, the balance between ECM anabolism which mainly include col2 and catabolism which mainly include Mmp13 is broken [11]. In intervertebral disc degeneration, Mmp13 expression quantity significantly increases in IDD as well as in arthritis [20]. As a result, Col2 expression quantity decreased and Mmp13 expression quantity increased in LSI group. The expression of Col2 and Mmp13 in the SSTZF group was significantly increased compared with LSI group. Therefore, SSTZF can maintain the normal integrity of cartilage endplate by restoring the metabolic balance of ECM, delaying the calcification and degeneration of the endplate.

During the IDD process, a large number of inflammatory factors, such as TNF-α and IL-1β, are produced in ECM. These cytokines promote matrix degradation, chemokines production and cell phenotypic change [48], leading to the dysmetabolism of ECM. In the previous report, inflammatory cytokines are closely related to NF-κB signaling pathway [49]. Also, it has been demonstrated that NF-κB is involved in IDD by regulating ECM degradation, oxidative stress, aging and cell death [31]. NF-κB exists as a dimer which is a member of the Rel protein family. Common Rel proteins are Rel A (p65), RelB, c-­Rel, p50 and p52 [50]. Rel A (p65) is one of the most widespread proteins in eukaryotic cells and has the most vital regulatory roles [51]. Also, phosphorylation and nuclear translocation of P65 are the classical activation of the NF-κB [52]. Therefore, the expression of p-P65 was detected. Compared with the model group, the expression of p-P65 in the SSTZF group was significantly reduced. These results suggest that SSTZF may delay IDD progression by inhibiting the activation of NF-κB. Previous studies have shown that SSTZF has a wide range of favorable therapeutic effects on orthopedic diseases, many of which are achieved through anti-inflammatory and regulation of internal stromal metabolic status. This is the first time that our results demonstrated that SSTZF can reduce inflammatory response and regulates stromal homeostasis in the treatment of IDD through inhibiting the activation of NF-κB.

However, the shortcoming of our present study is that we have not detected the molecular pharmacological mechanism of the active SSTZF. Investigating the possible biological processes of SSTZF may be beneficial for further research and help us using the SSTZF more effectively.

## Conclusions

In our study, the degeneration of LSI-induced mice’s tissue phenotype can be salvaged by SSTZF. The function of these, on the one hand, is that SSTZF can inhibit the ECM degradation pathway of intervertebral disc tissue and, on the other hand, is that SSTZF can inhibit the activation of NF-κB. Our results suggested that SSTZF can be used in the clinical as an alternative therapy for IDD.

## Data Availability

The data used to provide support for the results of this study can be obtained from the corresponding authors.

## References

[1] Bian Q, Jain A, Xu X (2016). Excessive activation of TGFβ by spinal instability causes vertebral endplate sclerosis. Sci Rep.

[2] Ni S, Ling Z, Wang X (2019). Sensory innervation in porous endplates by Netrin-1 from osteoclasts mediates PGE2-induced spinal hypersensitivity in mice. Nat Commun.

[3] Boos N, Weissbach S, Rohrbach H, Weiler C, Spratt KF, Nerlich AG (2002). Classification of age-related changes in lumbar intervertebral discs: 2002 Volvo Award in basic science. Spine (Phila Pa 1976).

[4] Wang WJ, Yu XH, Wang C (2015). MMPs and ADAMTSs in intervertebral disc degeneration. Clin Chim Acta.

[5] Sun Z, Yin Z, Liu C, Liang H, Jiang M, Tian J (2015). IL-1β promotes ADAMTS enzyme-mediated aggrecan degradation through NF-κB in human intervertebral disc. J Orthop Surg Res.

[6] Sun Z, Yin Z, Liu C, Tian J (2015). The changes in the expression of NF-KB in a degenerative human intervertebral disc model. Cell Biochem Biophys.

[7] Fang F, Jiang D (2016). IL-1β/HMGB1 signalling promotes the inflammatory cytokines release via TLR signalling in human intervertebral disc cells. Biosci Rep.

[8] Wang S, Liu C, Sun Z (2017). IL-1β increases asporin expression via the NF-κB p65 pathway in nucleus pulposus cells during intervertebral disc degeneration. Sci Rep.

[9] Ohba T, Haro H, Ando T (2009). TNF-alpha-induced NF-kappaB signaling reverses age-related declines in VEGF induction and angiogenic activity in intervertebral disc tissues. J Orthop Res.

[10] Zhang J, Wang X, Liu H (2019). TNF-α enhances apoptosis by promoting chop expression in nucleus pulposus cells: role of the MAPK and NF-κB pathways. J Orthop Res.

[11] Li Z, Zhang K, Li X (2018). Wnt5a suppresses inflammation-driven intervertebral disc degeneration via a TNF-α/NF-κB-Wnt5a negative-feedback loop. Osteoarthritis Cartilage.

[12] Chamoro M, de Luca K, Ozbulut O (2023). Association between clinical findings and the presence of lumbar spine osteoarthritis imaging features: a systematic review. Osteoarthritis Cartilage.

[13] Kennon JC, Awad ME, Chutkan N, DeVine J, Fulzele S (2018). Current insights on use of growth factors as therapy for Intervertebral disc degeneration. Biomol Concepts.

[14] Dionne CE, Dunn KM, Croft PR (2008). A consensus approach toward the standardization of back pain definitions for use in prevalence studies. Spine (Phila Pa 1976).

[15] Hartvigsen J, Hancock MJ, Kongsted A (2018). What low back pain is and why we need to pay attention. Lancet.

[16] Phillips KL, Jordan-Mahy N, Nicklin MJ, Le Maitre CL (2013). Interleukin-1 receptor antagonist deficient mice provide insights into pathogenesis of human intervertebral disc degeneration. Ann Rheum Dis.

[17] Wang X, Tan Y, Liu F (2023). Pharmacological network analysis of the functions and mechanism of kaempferol from Du Zhong in intervertebral disc degeneration (IDD). J Orthop Translat.

[18] Wang H, Jiang Z, Pang Z, Zhou T, Gu Y (2020). Acacetin alleviates inflammation and matrix degradation in nucleus pulposus cells and ameliorates intervertebral disc degeneration in vivo. Drug Des Devel Ther.

[19] Li J, Duan W, Chai S (2023). Wogonin, a bioactive ingredient from huangqi guizhi formula, alleviates discogenic low back pain via suppressing the overexpressed NGF in intervertebral discs. Mediators Inflamm.

[20] Vergroesen PP, Kingma I, Emanuel KS (2015). Mechanics and biology in intervertebral disc degeneration: a vicious circle. Osteoarthritis Cartilage.

[21] Hu S, Ge Q, Xia C (2020). Bushenhuoxue formula accelerates fracture healing via upregulation of TGF-β/Smad2 signaling in mesenchymal progenitor cells. Phytomedicine.

[22] Zhan JW, Li KM, Zhu LG (2022). Efficacy and safety of bushen huoxue formula in patients with discogenic low-back pain: a double-blind, randomized. Placebo-Controlled Trial Chin J Integr Med.

[23] Nijs J, D'Hondt E, Clarys P (2020). Lifestyle and chronic pain across the lifespan: An inconvenient truth?. Pm r.

[24] Lazaro-Pacheco D, Mohseni M, Rudd S, Cooper-White J, Holsgrove TP (2023). The role of biomechanical factors in models of intervertebral disc degeneration across multiple length scales. APL Bioeng.

[25] Speed C (2004). Low back pain. BMJ.

[26] Tessier S, Tran VA, Ottone OK (2020). TonEBP-deficiency accelerates intervertebral disc degeneration underscored by matrix remodeling, cytoskeletal rearrangements, and changes in proinflammatory gene expression. Matrix Biol.

[27] Xing H, Zhang Z, Mao Q (2021). Injectable exosome-functionalized extracellular matrix hydrogel for metabolism balance and pyroptosis regulation in intervertebral disc degeneration. J Nanobiotechnol.

[28] Roberts S, Caterson B, Menage J, Evans EH, Jaffray DC, Eisenstein SM (2000). Matrix metalloproteinases and aggrecanase: their role in disorders of the human intervertebral disc. Spine (Phila Pa 1976).

[29] Liu H, Pan H, Yang H (2015). LIM mineralization protein-1 suppresses TNF-α induced intervertebral disc degeneration by maintaining nucleus pulposus extracellular matrix production and inhibiting matrix metalloproteinases expression. J Orthop Res.

[30] Yang S, Li L, Zhu L (2019). Bu-Shen-Huo-Xue-Fang modulates nucleus pulposus cell proliferation and extracellular matrix remodeling in intervertebral disk degeneration through miR-483 regulation of Wnt pathway. J Cell Biochem.

[31] Zhang GZ, Liu MQ, Chen HW (2021). NF-κB signalling pathways in nucleus pulposus cell function and intervertebral disc degeneration. Cell Prolif.

[32] Bonnheim NB, Wang L, Lazar AA (2023). Deep-learning-based biomarker of spinal cartilage endplate health using ultra-short echo time magnetic resonance imaging. Quant Imaging Med Surg.

[33] Berg-Johansen B, Fields AJ, Liebenberg EC, Li A, Lotz JC (2018). Structure-function relationships at the human spinal disc-vertebra interface. J Orthop Res.

[34] Wong J, Sampson SL, Bell-Briones H (2019). Nutrient supply and nucleus pulposus cell function: effects of the transport properties of the cartilage endplate and potential implications for intradiscal biologic therapy. Osteoarthritis Cartil.

[35] Fields AJ, Ballatori A, Liebenberg EC, Lotz JC (2018). Contribution of the endplates to disc degeneration. Curr Mol Biol Rep.

[36] Li X, Yang S, Han L, Mao K, Yang S (2020). Ciliary IFT80 is essential for intervertebral disc development and maintenance. Faseb J.

[37] Bydon M, De la Garza-Ramos R, Macki M, Baker A, Gokaslan AK, Bydon A (2014). Lumbar fusion versus nonoperative management for treatment of discogenic low back pain: a systematic review and meta-analysis of randomized controlled trials. J Spinal Disord Tech.

[38] Dolor A, Sampson SL, Lazar AA, Lotz JC, Szoka FC, Fields AJ (2019). Matrix modification for enhancing the transport properties of the human cartilage endplate to improve disc nutrition. PLoS ONE.

[39] Wang XF, Zhang AP, Sun ZY, Liu C, Kuang LH, Tian JW (2017). Expression of NF-κB in a degenerative human intervertebral disc model. Zhonghua Yi Xue Za Zhi.

[40] Zhongyi S, Sai Z, Chao L, Jiwei T (2015). Effects of nuclear factor kappa B signaling pathway in human intervertebral disc degeneration. Spine (Phila Pa 1976).

[41] Li Z, Wang X, Pan H (2017). Resistin promotes CCL4 expression through toll-like receptor-4 and activation of the p38-MAPK and NF-κB signaling pathways: implications for intervertebral disc degeneration. Osteoarthritis Cartilage.

[42] Chen J, Liu GZ, Sun Q (2019). Protective effects of ginsenoside Rg3 on TNF-α-induced human nucleus pulposus cells through inhibiting NF-κB signaling pathway. Life Sci.

[43] Miao M, Gao M, Li T (2020). Tandem mass tag-based proteomic analysis reveals the treatment mechanism of Bushen Huoxue Formula on psychological stress-induced premature ovarian insufficiency. J Ethnopharmacol.

[44] Ji WF, Shi WF, Chen L (2012). Experimental study on invigorating kidney and activating blood on preventing and curing SD rats with knee osteoarthritis. Zhongguo Gu Shang.

[45] Wang PE, Zhang L, Ying J (2018). Bushenhuoxue formula attenuates cartilage degeneration in an osteoarthritic mouse model through TGF-β/MMP13 signaling. J Transl Med.

[46] Zhou Z, Tian FM, Wang P (2015). Alendronate prevents intervertebral disc degeneration adjacent to a lumbar fusion in ovariectomized rats. Spine (Phila Pa 1976).

[47] Rade M, Määttä JH, Freidin MB, Airaksinen O, Karppinen J, Williams FMK (2018). Vertebral endplate defect as initiating factor in intervertebral disc degeneration: strong association between endplate defect and disc degeneration in the general population. Spine (Phila Pa 1976).

[48] Risbud MV, Shapiro IM (2014). Role of cytokines in intervertebral disc degeneration: pain and disc content. Nat Rev Rheumatol.

[49] Bowles RD, Mata BA, Bell RD (2014). In vivo luminescence imaging of NF-κB activity and serum cytokine levels predict pain sensitivities in a rodent model of osteoarthritis. Arthritis Rheumatol.

[50] Hayden MS, Ghosh S (2008). Shared principles in NF-kappaB signaling. Cell.

[51] Wang Y, Wang L, Wen X (2019). NF-κB signaling in skin aging. Mech Ageing Dev.

[52] Jimi E, Katagiri T (2022). Critical roles of NF-κB signaling molecules in bone metabolism revealed by genetic mutations in osteopetrosis. Int J Mol Sci.

